# Significance of systolic-phase imaging on full-phase ECG-gated CT angiography to detect intimal tears in aortic dissection

**DOI:** 10.1007/s00380-022-02093-0

**Published:** 2022-05-15

**Authors:** Satoru Yanagaki, Atsuro Masuda, Hideki Ota, Masatoshi Kojima, Takashi Hattori, Wahei Mihara, Kei Takase, Takuya Ueda

**Affiliations:** 1 Departments of Diagnostic Radiology, Tohoku University Graduate School of Medicine, Seiryo 1-1, Sendai, 980-8574 Japan; 2 Departments of Radiology, Seikeikai Chiba Medical Center, 1-7-1, Minami-cho, Chuo-ku, Chiba, 260-0842 Japan; 3 Departments of Cardiovascular Surgery, Seikeikai Chiba Medical Center, 1-7-1, Minami-cho, Chuo-ku, Chiba, 260-0842 Japan; 4 Departments of Clinical Imaging, Tohoku University Graduate School of Medicine, Seiryo 1-1, Sendai, 980-8574 Japan

**Keywords:** Aortic dissection, Intramural hematoma, Retrospective ECG-gated CTA, Intimal tear, Ulcer-like projection

## Abstract

**Purpose:**

For patients with aortic dissection (AD) and intramural hematoma (IMH), the optimal cardiac phase to detect intimal tears (IT) and ulcer-like projections (ULP) on retrospective electrocardiogram (ECG)-gated computed tomography angiography (CTA) remains unclear. The purpose of this study was to compare the accuracy of retrospective ECG-gated CTA for detecting IT in AD and ULP in IMH between each cardiac phase.

**Materials and methods:**

A total of 75 consecutive patients with AD and IMH of the thoracic aorta were enrolled in this single-center retrospective study. The diagnostic performance to detect IT and ULP in the thoracic aortic regions (including the ascending aorta, aortic arch, and proximal and distal descending aorta) was compared in each cardiac phase on retrospective ECG-gated CTA.

**Results:**

In the systolic phase (20%), the accuracy, sensitivity, and specificity to detect IT in AD was 64% (95% confidence interval [CI] 56–72%), 69% (95%CI 60–78%), and 25% (95%CI 3.3–45%), respectively. In the diastolic phase (70%), the accuracy, sensitivity, and specificity to detect IT in AD was 52% (95%CI 43–60%), 52% (95%CI 42–61%), and 50% (95%CI 25–75%), respectively. The accuracy to detect IT in AD on ECG-gated CTA was significantly higher in the systolic phase than that in the diastolic phase (*P* = 0.025). However, there were no differences in the accuracy (83%; 95%CI 78–89%), sensitivity (71%; 95%CI 62–80%), or specificity (100%; 95%CI 100%) to detect ULP in IMH between the cardiac cycle phases.

**Conclusion:**

Although it is currently recommended for routine diagnosis of AD and IMH, single-diastolic-phase ECG-gated CTA has risk to miss some IT in AD that are detectable in the systolic phase on full-phase ECG-gated CTA. This information is critical for determining the optimal treatment strategy for AD.

## Introduction

Aortic dissection (AD) is a life-threatening disease with an estimated annual incidence of 6–10 cases per 100,000 people and a mortality rate of 25–30% [1–3]. Recent guidelines for AD have emphasized the importance of detecting intimal tears (IT) in AD and ulcer-like projections (ULP) in intramural hematoma (IMH) [4–6], because this affects the treatment strategy decided at initial assessment in patients with acute AD in the emergency department [7, 8].

Electrocardiogram (ECG)-gated computed tomography angiography (CTA) was originally used to improve image quality in cardiac disease by reducing motion artifacts caused by pulsatile motion during the cardiac cycle [9]. Previous studies have also reported the utility of ECG-gated CTA for assessment of AD, with better image quality and fewer motion artifacts than for non-ECG-gated CTA [10, 11]. Although prospective single-diastolic-phase ECG-gated CTA is typically recommended to reduce radiation exposure, it remains unclear whether the diastolic phase is the optimal cardiac phase for detecting IT in AD and ULP in IMH, regardless of the presence of motion artifacts.

Yanagaki et al. reported that full-phase ECG-gated CTA was more accurate for detecting IT in AD and ULP in IMH than for non-gated CTA and single-diastolic-phase ECG-gated CTA, and suggested that single-diastolic-phase ECG-gated CTA may overlook IT and ULP that can be detected by full-phase retrospective ECG-gated CTA [12]. Nevertheless, the optimal phase for detecting IT and ULP during the cardiac cycle in retrospective ECG-gated CTA remains unclear.

The purpose of this study was to compare the accuracy for detecting IT in AD and ULP in IMH between the cardiac phases using retrospective ECG-gated CTA.

## Materials and methods

### Study participants

**Fig. 1:**
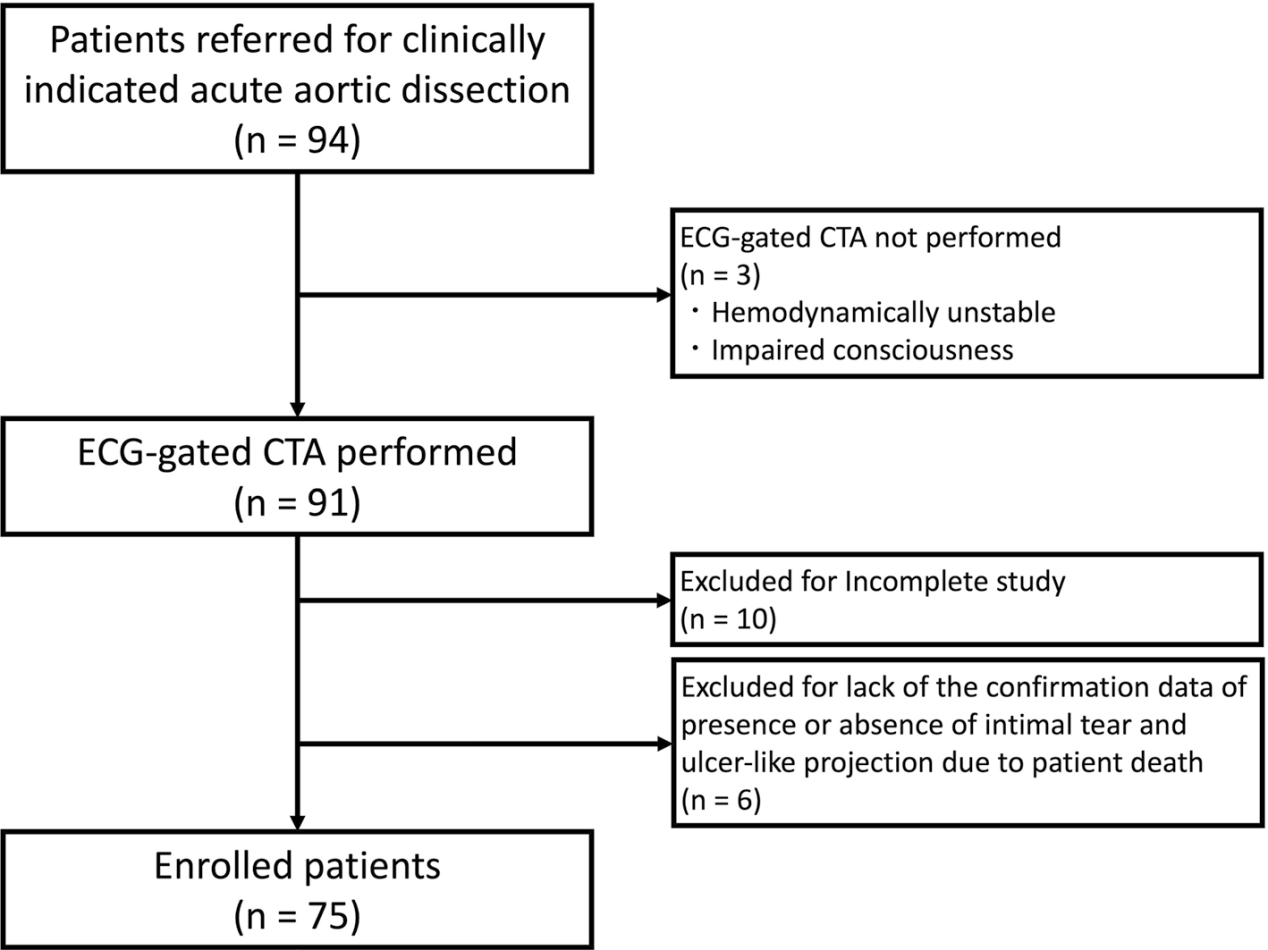
Flow diagram of the study participants

This was a retrospective cohort study that included patients treated in a single institution. The institutional review board approved this study and waived the requirement for informed consent. Consecutive patients who were referred to the emergency department in our hospital from January 2016 to December 2018 and were diagnosed with AD and IMH were included. Of 94 included patients, 75 patients who underwent ECG-gated CTA were included in this study (Fig. 1). Ten patients were excluded for incomplete study data and six patients were excluded for a lack of confirmation data on the presence or absence of IT and ULP because of patient death.

### CT angiography protocol

All CT examinations were performed using one of two CT scanners (SOMATOM Definition AS + or SOMATOM Definition Flash; Siemens Medical Solutions, Forchheim, Germany). The patients were scanned with a tube voltage of 120 kV and a tube current of 180 mAs/rotation. The pitch was set to 0.2 with a gantry rotation time of 0.33 s in ECG-gated CTA and to 1.2 with a gantry rotation time of 0.28 s in non-ECG-gated CTA. The collimation was 128 × 0.6 mm. Dose modulation was not performed.

After a scout view was obtained, unenhanced images were acquired. A bolus-triggering technique was used in all patients. Contrast medium (Iopamiron 370; Bayer Health care, Osaka, Japan; or Oiparomin 370; Fuji Pharmaceutical Kogyo Co., Ltd, Tokyo, Japan) was initially injected using a split-bolus injection technique at 20 mgI/kg/s for 20 s, followed by 10 mgI/kg/sec for 10 s. The thoracic ECG-gated CTA in the aortic contrast-enhanced phase was acquired. The scan delay was determined by automated bolus triggering to detect when the enhancement within the ascending aorta exceeded 100 HU. Immediately after the thoracic ECG-gated CTA, whole body non-ECG-gated CTA in the aortic contrast-enhanced phase was acquired. The approximate time for each scan was 6 s in the first phase and 4 s in the second phase. Beta blockers were not administered to control heart rate.

### Image reconstruction

For each patient, ECG-gated CTA data were reconstructed into ten series of images at 0–90% with 10% interval increments of the R-R interval in the cardiac cycle. Images were reconstructed with a 1-mm interval using the soft tissue convolution kernel I36f. All datasets were transferred to a workstation (Osirix v.10.0.3; Pixmeo SARL, Bernex, Switzerland) for image assessment.

### Image analysis

The ECG-gated CTA images in each cardiac cycle were assessed independently in random order by two radiologists (S.Y and A.M.) with 5 and 10 years of experience in cardiovascular imaging, respectively. Both radiologists were blinded to the patients’ clinical data and the acquired cardiac phase of the images. In case with discrepancy in the assessment, we added third radiologist was and held a discussion. Evaluation was performed in a random order for each phase image of each patient.

**Fig. 2:**
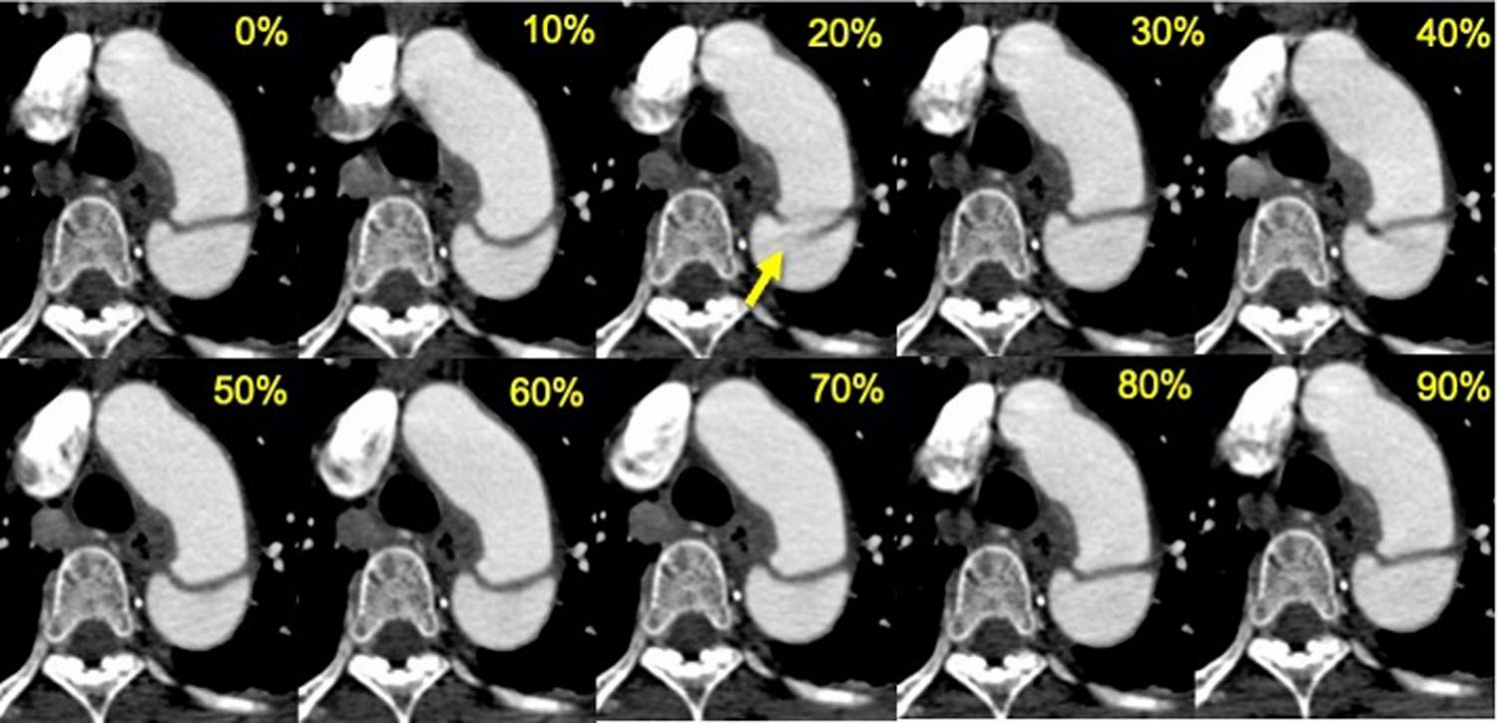
Images comparing retrospective ECG-gated CT angiography at each phase of the R-R interval in a 53-year-old man with acute aortic dissection

**Fig. 3:**
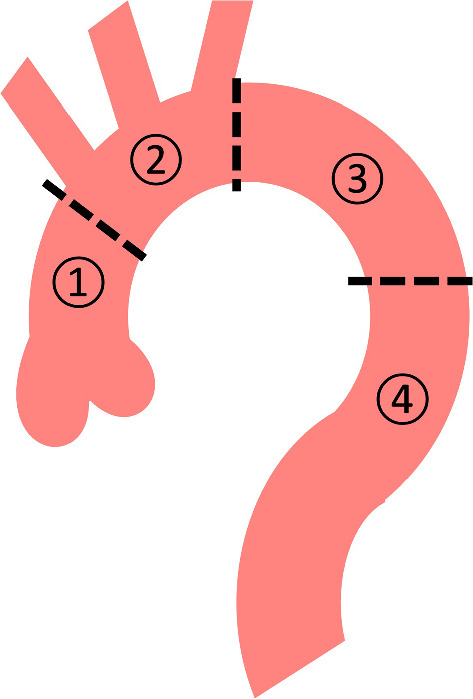
Segmentation of the thoracic aorta for assessment. (1) Ascending aorta, (2) aortic arch, (3) proximal descending aorta, and (4) distal descending aorta. The ascending aorta was defined from the origin of the right coronary artery to the origin of the brachiocephalic artery (zone 0 in thoracic endovascular aortic replacement [TEVAR]). The aortic arch was defined from the origin of the brachiocephalic artery to the origin of the left subclavian artery (zones 1–2 in TEVAR). The proximal descending aorta was defined from the origin of the left subclavian artery to the level of the pulmonary bifurcation (zone 3 in TEVAR). The distal descending aorta was defined from the level of the pulmonary bifurcation to the aortic hiatus of the diaphragm (zone 4 in TEVAR)

The observer evaluated the presence or absence of defined lesions in each cardiac phase using the following criteria. IT was defined as a defect of the intimal flap with communication between the true and false lumen in AD (Fig. 2) [13, 14]. ULP was defined as a localized pouch filled with contrast medium protruding into the thrombosed false lumen of the aorta [7, 13]. The thoracic aorta was divided into four segments: the ascending aorta, aortic arch, proximal descending aorta, and distal descending aorta (Fig. 3). The presence of IT and ULP was independently assessed in each segment of the thoracic aorta on ECG-gated CTA images. For this assessment, multiplanar reformations of each dataset were reconstructed if needed.

During the cardiac cycle, the 0–40% phase and the 50–90% phase in the R-R interval were defined as the systolic phase and diastolic phase, respectively [15]. We also defined the 20% and 70% phases for the representative systolic and diastolic phases, respectively.

### Confirmation of the presence of IT and ULP

The presence and location of IT and ULP were confirmed by the surgical records for patients who underwent surgery, by angiographic findings for patients who underwent angiography, or by follow-up CT for patients who did not undergo surgery or angiography—we judged the lesions to be present when the lesions were detected more than three times on follow-up CT. Follow-up CT was typically performed at 1 week, 2 weeks, 1 month, and 3 months after admission.

### Statistical analysis

The accuracy, sensitivity, and specificity to detect IT and ULP were calculated using the following formulas: accuracy = (true positive + true negative) / (true positive + true negative + false positive + false negative); sensitivity = true positive / (true positive + false negative); specificity = true negative / (true negative + false positive). For patient-based analysis, assessment was considered as a true positive when the lesion was correctly detected in any part of the thoracic aorta of each patient. For location-based analysis, the accuracy, sensitivity, and specificity were calculated on each anatomical region. All statistical analyses were performed using commercially available software (JMP Pro v14.0.0; SAS institute Inc., Cary, NC, USA). The McNemar test was used to compare the accuracy between the systolic phase (20%) and the diastolic phase (70%). A *P-*value < 0.05 was considered statistically significant.

The interobserver agreement between the two radiologists regarding the judgment of IT and ULP was evaluated using κ statistics. A κ value of more than 0.81 corresponded to excellent interobserver agreement, while values of 0.61–0.80 corresponded to good agreement.

## Results

### Study participants

A total of 75 patients (54 men, 21 women; mean age, 66.9 ± 12.6 years; age range, 32–94 years) underwent successful CTA without any complications. The demographic characteristics are shown in Table 1. In patients with AD, 29 IT in 92 dissected segments from a total of 132 segments (4 anatomical segments for each of 33 patients) were detected. A total of 24 ULP from 42 patients with IMH were detected. There was no patient who had additional surgery and endovascular therapy and had anatomical change in follow-up period.

**Table 1:** Patient characteristics

Mean age (*y*)	66.9 ± 12.6
*Sex*	
Male	54 (77)
Female	21 (23)
*Stanford classification*	
Type A	23 (31)
Type B	52 (69)
*Disease type*	
Aortic dissection	33 (44)
Intramural hematoma	42 (56)
*Surgery*	
Ascending aorta replacement	20 (27)
Total aortic arch replacement (TAR)	5 (7)
TAR + Open Stentgrafting	8 (11)
Thoracic endovascular aortic repair (TEVAR)	7 (9)
Medical treatment without surgery nor (TEVAR)	35 (47)

Continuous variables are expressed as mean ± standard deviation

Categorical variables are expressed as *n* (%)

### Patient-based analysis to detect IT in AD and ULP in IMH

**Fig. 4:**
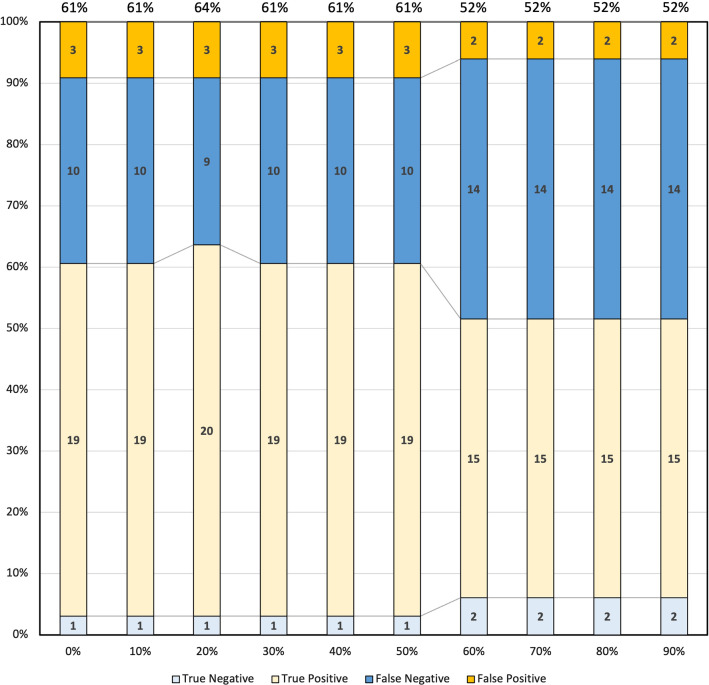
Accuracy for detection of intimal tears in aortic dissection (*n* = 33)

The accuracy, sensitivity, and specificity to detect IT in AD and ULP in IMH in each cardiac phase are shown in Table 2. The number of true positives, true negatives, false positives, and false negatives to detect IT in AD are shown in Fig. 4. In the systolic phase (20%), the accuracy, sensitivity, and specificity to detect IT in AD were 64% (95% confidence interval [CI] 56–72%), 69% (95%CI 60–78%), and 25% (95%CI 3.3–45%), respectively. In the diastolic phase (70%), the accuracy, sensitivity, and specificity to detect IT in AD were 52% (95%CI 43–60%), 52% (95%CI 42–61%), and 50% (95%CI 25–75%), respectively. The accuracy to detect IT on ECG-gated CTA was significantly higher in the systolic phase than that in the diastolic phase (*P* = 0.025). The accuracy, sensitivity, and specificity to detect ULP in IMH were 83% (95%CI 78–89%), 71% (95%CI 62–80%), and 100% (95%CI 100%), respectively, in all phases during the cardiac cycle. All interobserver agreements were excellent (κ = 0.86).

**Table 2:** Accuracy, sensitivity, and specificity for detecting intimal tears in aortic dissections and ulcer-like projections in intramural hematomas

Aortic dissection (*n* = 33)										
Cardiac cycle	0%	10%	20%	30%	40%	50%	60%	70%	80%	90%
Accuracy	61 (69, 52)	61 (69, 52)	64 (72, 56)	61 (69, 52)	61 (69, 52)	61 (69, 52)	52 (60, 43)	52 (60, 43)	52 (60, 43)	52 (60, 43)
Sensitivity	66 (74, 57)	66 (74, 57)	69 (78, 60)	66 (74, 57)	66 (74, 57)	66 (74, 57)	52 (61, 42)	52 (61, 42)	52 (61, 42)	52 (61, 42)
Specificity	25 (47, 3.3)	25 (47, 3.3)	25 (47, 3.3)	25 (47, 3.3)	25 (47, 3.3)	25 (47, 3.3)	50 (75, 25)	50 (75, 25)	50 (75, 25)	50 (75, 25)
Intramural hematoma (*n* = 42)										
Accuracy	83 (89, 78)	83 (89, 78)	83 (89, 78)	83 (89, 78)	83 (89, 78)	83 (89, 78)	83 (89, 78)	83 (89, 78)	83 (89, 78)	83 (89, 78)
Sensitivity	71 (80, 62)	71 (80, 62)	71 (80, 62)	71 (80, 62)	71 (80, 62)	71 (80, 62)	71 (80, 62)	71 (80, 62)	71 (80, 62)	71 (80, 62)
Specificity	100 (100, 100)	100 (100, 100)	100 (100, 100)	100 (100, 100)	100 (100, 100)	100 (100, 100)	100 (100, 100)	100 (100, 100)	100 (100, 100)	100 (100, 100)

Data are presented as percentages with 95% confidence intervals in parentheses, unless otherwise indicated

### Location-based analyses to detect IT in AD

**Fig. 5:**
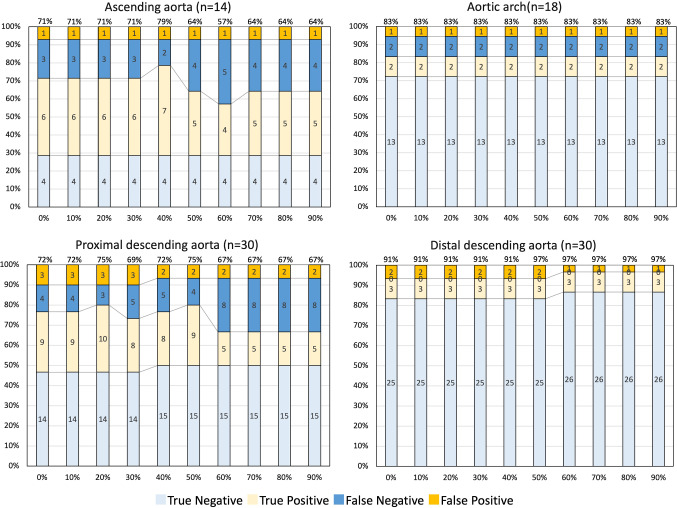
Accuracy for detection of intimal tears in aortic dissection at each anatomical region

For the location-based analysis, the accuracy, sensitivity, and specificity to detect IT in AD in each cardiac phase are shown in Table 3. The number of true positives, true negatives, false positives, and false negatives to detect IT in AD in each anatomical segment are shown in Fig. 5. In the ascending aorta, the accuracy to detect IT in AD was 71% (95%CI 59–84%) in the systolic phase and 64% (95%CI 51–77%) in the diastolic phase. There was no significant difference in accuracy between the systolic phase and the diastolic phase (*P* = 0.32). In the aortic arch, the accuracy, sensitivity, and specificity to detect IT in AD were 84% (95%CI 75–92%), 50% (95%CI 25–75%), and 93% (95%CI 86–100%), respectively, in all cardiac phases. In the proximal descending aorta, the accuracy to detect IT in AD was 80% (95%CI: 73%–87%) in the systolic phase and 67% (95%CI: 58%–75%) in the diastolic phase. The accuracy to detect IT in the ascending aorta was significantly higher in the systolic phase than that in the diastolic phase (*P* = 0.046). In the distal descending aorta, the accuracy and specificity to detect IT in AD was 91% (95%CI 93–100%) in the systolic phase and 97% (95%CI 93–100%) in the diastolic phase. There was no significant difference in accuracy between the systolic phase and the diastolic phase (*P* = 0.32).

**Table 3:** Accuracy, sensitivity, and specificity for detecting intimal tears in aortic dissections at each anatomical segment

Ascending aorta (*n* = 14)										
Cardiac cycle	0%	10%	20%	30%	40%	50%	60%	70%	80%	90%
Accuracy	71 (84, 59)	71 (84, 59)	71 (84, 59)	71 (84, 59)	79 (90, 68)	64 (77, 51)	57 (70, 44)	64 (77, 51)	64 (77, 51)	64 (77, 51)
Sensitivity	66 (82, 51)	66 (82, 51)	66 (82, 51)	66 (82, 51)	78 (92, 64)	56 (72, 39)	44 (61, 28)	56 (72, 39)	56 (72, 39)	56 (72, 39)
Specificity	80 (98, 62)	80 (98, 62)	80 (98, 62)	80 (98, 62)	80 (98, 62)	80 (98, 62)	80 (98, 62)	80 (98, 62)	80 (98, 62)	80 (98, 62)
Aortic arch (*n* = 18)										
Accuracy	84 (92, 75)	84 (92, 75)	84 (92, 75)	84 (92, 75)	84 (92, 75)	84 (92, 75)	84 (92, 75)	84 (92, 75)	84 (92, 75)	84 (92, 75)
Sensitivity	50 (75, 25)	50 (75, 25)	50 (75, 25)	50 (75, 25)	50 (75, 25)	50 (75, 25)	50 (75, 25)	50 (75, 25)	50 (75, 25)	50 (75, 25)
Specificity	93 (100, 86)	93 (100, 86)	93 (100, 86)	93 (100, 86)	93 (100, 86)	93 (100, 86)	93 (100, 86)	93 (100, 86)	93 (100, 86)	93 (100, 86)
Proximal descending aorta (*n* = 30)										
Accuracy	77 (84, 69)	77 (84, 69)	80 (87, 73)	73 (81, 65)	77 (84, 69)	80 (87, 73)	67 (75, 58)	67 (75, 58)	67 (75, 58)	67 (75, 58)
Sensitivity	69 (82, 56)	69 (82, 56)	77 (89, 65)	62 (77, 48)	62 (77, 48)	69 (82, 56)	38 (52, 25)	38 (52, 25)	38 (52, 25)	38 (52, 25)
Specificity	82 (92, 73)	82 (92, 73)	82 (92, 73)	82 (92, 73)	88 (96, 80)	88 (96, 80)	88 (96, 80)	88 (96, 80)	88 (96, 80)	88 (96, 80)
Distal descending aorta (*n* = 30)										
Accuracy	93 (98, 89)	93 (98, 89)	93 (98, 89)	93 (98, 89)	93 (98, 89)	93 (98, 89)	97 (100, 93)	97 (100, 93)	97 (100, 93)	97 (100, 93)
Sensitivity	100	(100, 100)	(100, 100)	(100, 100)	(100, 100)	(100, 100)	(100, 100)	(100, 100)	(100, 100)	(100, 100)
Specificity	93 (98, 88)	93 (98, 88)	93 (98, 88)	93 (98, 88)	93 (98, 88)	93 (98, 88)	96 (100, 93)	96 (100, 93)	96 (100, 93)	96 (100, 93)

Data are presented as percentages with 95% confidence intervals in parentheses, unless otherwise indicated

## Discussion

Recent guidelines for AD have advocated the importance of detecting IT including entry/re-entry in AD and ULP in IMH [4–6]. Several studies have emphasized importance of detection of IT of AD at initial surgery, which is a key indication for aortic arch replacement rather than simple ascending aortic replacement—Residual IT on the aortic arch is a risk factor for reoperation of simple ascending aortic replacement [16]. The presence of ULP in IMH is also an important factor for determining the surgical indication of complicated IMH [17–19]. In patients who are indicated thoracic endovascular aortic repair, complete sealing of IT and ULP is necessary to achieve a satisfactory treatment outcome [14, 20, 21]. Such a change in the treatment strategy makes accurate detection of IT in AD and ULP in IMH of even greater clinical importance [22].

ECG-gated CTA uses either prospective or retrospective data acquisition. Prospective ECG-gated CTA only acquires image data at a predetermined single phase of the cardiac cycle. By contrast, retrospective ECG-gated CTA acquires image data during the entire cardiac cycle, which enables the reconstruction of images from any cardiac cycle phase [23–25]. Numerous studies have reported the utility of single-diastolic-phase ECG-gated CTA for AD diagnosis, based on its excellent image quality and reduced image noise and motion artifacts [15, 26, 27]. Nevertheless, Yanagaki et al. reported the significance of full-phase retrospective ECG-gated CTA in detecting the IT in AD and the ULP in IMH compared with non-ECG-gated CTA and single-diastolic-phase ECG-gated CTA [12].

Retrospective ECG-gated CTA has the disadvantage of increased radiation exposure in comparison with prospective ECG-gated CTA [11, 25]. However, the potential improvement in the detection of the IT and ULP may outweigh the disadvantage of increased radiation, as treatment failure may directly lead to patient death in such an emergency.

Our study showed that ECG-gated CTA had a significantly higher accuracy for detecting IT in AD in the systolic phase than in the diastolic phase. The previous study using computational fluid dynamics reported that the rupture positions in aortic dissection corresponded to the area of maximum hemodynamic stress and analysis of magnetic resonance-derived metrics demonstrated that maximum false lumen pressure in systolic phase correlated with aortic growth [28, 29]. Therefore, in the systolic phase, higher hemodynamic stress and pressure is applied to the aortic wall compared to the diastolic phase which may cause reveal IT. Thus, single-diastolic-phase ECG-gated CTA may miss an IT that would be detected in the systolic phase by full-phase ECG-gated CTA. In patients with Stanford type A AD, failure to detect an IT of the aortic arch represents a missed opportunity for appropriate graft replacement of the total aortic arch [16]. Furthermore, in patients with Stanford type B AD, failure to detect IT of the aortic arch would result in incomplete sealing of the IT at endovascular aortic repair [30–32].

We found no differences in detection of ULP in IMH between the cardiac cycles. This may be because the flap motion in IMH is not as large as that in AD because of thrombosis of the false lumen. The optimal systolic phases to detect an IT in AD also differed with IT location. Thus, in emergency medicine, all cardiac cycle phases should be assessed to maximize the detectability of IT, which is important for determining the optimal treatment strategy.

Our study has three limitations. First, there are potential problems when using reference data in the following situations. (1) Although surgical confirmation of IT and ULP is the gold standard to determine the presence or absence of IT and ULP, surgery was performed only in 33 (44%) patients. (2) IT and ULP distal to the descending aorta may be missed during surgery because of difficulties in visualizing the surgical field. In this case, the detection of IT and ULP using image-bases can cause false positives. (3) In patients who did not receive surgery or angiography, the presence of IT and ULP is determined by follow-up-based confirmation. Because IT and ULP that existed in the early stages of onset may heal and disappear during the course of follow-up [33], this may also cause false positives. Second, our study excluded patients who died and could not undergo ECG-gated CTA, which may have caused a patient selection bias. Third, this was a single-facility study involving a small number of patients and statistical differences were small. A multicenter study needs to be considered in the future to validate the applicability of our findings.

## Conclusion

Our study demonstrated that when using full-phase ECG-gated CTA, some IT detected in the systolic phase are not detected in the diastolic phase. At initial assessment of suspicious acute aortic disease, full-phase ECG-gated CTA is recommended to maximize the detectability of IT, which will directly affect the treatment strategy in the emergency situation.

## Abbreviations

IT: Intimal tear
AD: Aortic dissection
ULP: Ulcer-like projection
IMH: Intramural hematoma
ECG: Electrocardiogram
